# Tree-rings analysis to reconstruct atmospheric mercury contamination at a historical mining site

**DOI:** 10.3389/fpls.2023.1260431

**Published:** 2023-10-13

**Authors:** Davide Baroni, Stefania Ancora, Jürgen Franzaring, Stefano Loppi, Fabrizio Monaci

**Affiliations:** ^1^ Department of Physical Sciences, Earth and Environment, University of Siena, Siena, Italy; ^2^ Institute of Landscape and Plant Ecology, University of Hohenheim, Stuttgart, Germany; ^3^ Department of Life Sciences, University of Siena, Siena, Italy

**Keywords:** tree rings, mercury, thallium, lead, mining, contaminated site, reclamation, biomonitoring

## Abstract

Mercury (Hg) is a global environmental concern due to its toxicity (especially high in methylated form) and the long-range distribution of its gaseous elemental form (GEM). Hg-contaminated areas, such as abandoned mining sites, pose intrinsic difficulties for their management and heavy monitoring costs. In these environments, plant-based solutions may play a key role in the ecosystem quality assessment and support remediation strategies, combining reliability and cost-effectiveness. In this study, we adopted a biomonitoring approach by using tree rings of four different species collected in the proximity of the mining-metallurgical area of Abbadia San Salvatore, central Italy, a major former Hg mining district whose reclamation is currently in progress. Our dendrochemical analysis was aimed at identifying the historical changes of local atmospheric Hg contamination and at singling out, for the first time in the study area, other potentially toxic elements (PTEs) associated with the past mining activity. Collected cores dated back to early as 1940 and provided the temporal patterns of atmospheric Hg emission vs the produced liquid quantities, so reconstructing the historical impact of the mining site on nearby terrestrial ecosystems and resident human population. Current GEM contamination was found about twenty times lower than that of the fully operational mine periods. From a first survey on other PTEs, thallium (Tl) and lead (Pb) appeared to be potentially associated with the mining activity, thus suggesting new working assumptions for further dendrochemical analyses and for the inclusion of Pb in human biomonitoring surveys of the Mt. Amiata area, actually not present in the control list. The results prompt a more thorough assessment by tracking for a longer time span a critical site that is an ideal open-field lab to study the ecophysiology of different tree species in relation to environmental behavior of PTEs for better-assessing wildlife and human exposures.

## 1 Introduction

Mercury (Hg) is especially capable of pervasive environmental impacts, being toxic to humans and globally distributed through its atmospheric predominant (approx. 95%) and long-lasting (6–18 months) gaseous elemental form (GEM; Gworek et al., 2017). The inputs to terrestrial ecosystems are mediated by wet and dry deposition, the latter distinguished into deposition to non-vegetation land surfaces (soils, snow and water) and direct vegetation uptake of Hg^2+^ (10%) and Hg^0^ (90%). GEM assimilation by vegetation is considered the predominant process (1,180–1,410 Mg/yr), thus representing the main single terrestrial removal pathway of atmospheric Hg (Zhou et al., 2021).

With the increasing reckoning of the critical importance of vegetation in terrestrial Hg cycling, the distribution patterns in plant parts (e.g., leaves, litterfall, woody tissues) of different functional groups (e.g., grassland plants, shrubs, trees; but also lichens and mosses) has become a research priority to investigate Hg dynamics globally (Zhou and Obrist, 2021). Among these biomonitoring methods, tree rings analysis has a special use for understanding how Hg emissions to the atmosphere have changed over decades or even centuries (Clackett et al., 2018; McLagan et al., 2022). In fact, concentrations in tree rings serve as proxies for estimating the magnitude of a plant’s Hg assimilation through time, useful for the retrospective tracking of air Hg contamination at a specific area (Gustin et al., 2022).

In this study, we used tree rings as archives of atmospheric Hg emissions at Abbadia San Salvatore, in Mt. Amiata, central Italy, a former Hg mining district established since the antiquity (900-700 BC) on a vast belt of Hg mineralization (cinnabar, HgS), and one of the main global manufacturers of liquid Hg for much of the 20^th^ century. Hg production in Abbadia S.S. officially ceased in 1984, and since then the site, and especially the smelting plants therein, have continued to be significant sources of GEM at the global scale (80-150 kg GEM/year; McLagan et al., 2019). Through analysis of tree rings from four different species, we aimed to identify the temporal patterns of atmospheric Hg emission vs the produced quantities to reconstruct the historical impact of the mining site on nearby terrestrial ecosystems and resident population. In a first attempt to single out possible tracers of local elemental contamination, other potentially toxic elements (PTEs) that could be associated with the mining-metallurgical activities in Abbadia S.S. were also included in the analysis.

## 2 Method

**Figure 1:**
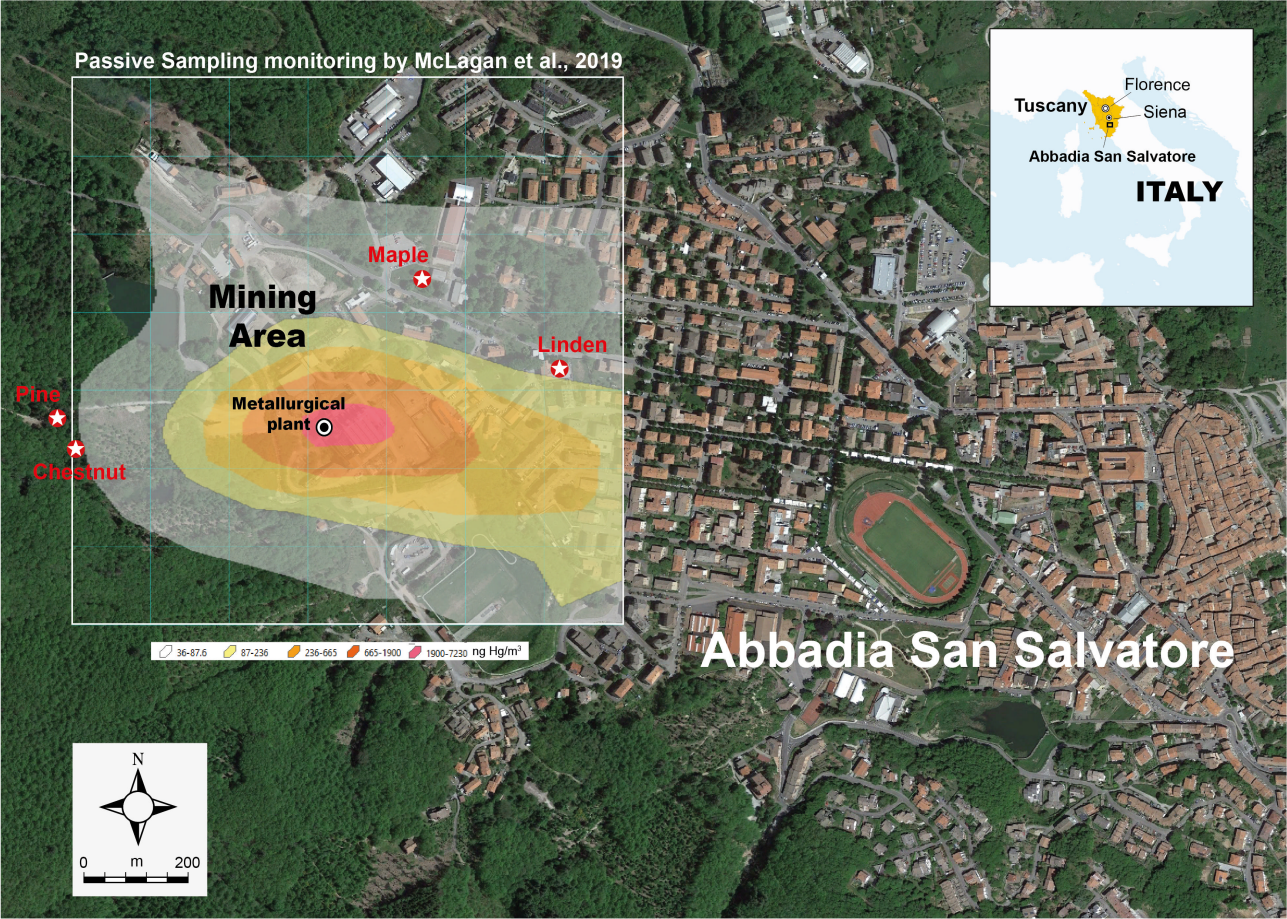
Sampling sites of tree cores in the area of the Mine of Abbadia San Salvatore (Google Earth) and superimposed representation of the distribution of time-weighted average concentrations of GEM as reported by McLagan et al., 2019.

GEM concentrations map of the mining site of Abbadia S.S. and the nearby urban area, recently produced by using passive samplers (McLagan et al., 2019), was taken in this study as the main reference for sampling design. Sampling sites (**Figure 1**) were selected considering GEM dispersal from the metallurgical plant, the hotspot of contamination in the area (McLagan et al., 2019). From the latter, the selected sampling sites were at a distance of 340-500 m in zones where it was possible to locate trees of four species: maple (*Acer pseudoplatanus*), pine (*Pinus nigra*), chestnut (*Castanaea sativa*) and linden (*Tilia cordata*).

Tree cores were collected in October 2022 using a 400 mm long Haglöf increment borer, having a 5.15 mm diameter. Two cores from each tree were taken from the east and west sides of the trunk. During sampling, cleaning actions were put in place to avoid any potential contamination (e.g. operators were always using latex gloves, and the borer was washed with Milli-Q water and blown drying with canned air prior to use and after each sampling). After collection, each core was stored in a 600 mm long 10 mm diameter PVC pipe which was sealed with a rubber socket cap on each end.

In the laboratory, samples were stored in a freezer at -20°C and later dried at room temperature for a week. After cross-dating the cores with standard dendrochronological methods, ring widths were measured with a dissecting Leica microscope; starting from the outside towards the center, the cores were finally cut into 3-year segments for linden and into 5-year segments for the other species (3-year the innermost chestnut segment) and lyophilized in a LIO 5Pascal (t = 24 h, T = -49,5°C, P = 0,125 mbar) prior to analysis to achieve complete dryness. Total concentrations of Hg and 13 other elements, namely arsenic (As), boron (B), cadmium (Cd), cobalt (Co), chromium (Cr), copper (Cu), manganese (Mn), nickel (Ni), lead (Pb), antimony (Sb), thallium (Tl), vanadium (V) and zinc (Zn) of each core segment were determined at the Core Facility - CFH of the University of Hohenheim by using an inductively coupled plasma mass spectrometer (ICP-MS, NexION 300X, PerkinElmer) after mineralization with HNO_3_ and H_2_O_2_ in a microwave digestion unit (UltraCLAVEIII, MLS GmbH). Before ICP-MS analysis, L-Cysteine was added to all standards, samples and rinse solution to prevent the memory effects of Hg. To fulfill quality control requirements, routine procedural blanks, rhodium ICP standard solution as internal standards together with certified reference materials (1547 “Peach Leaves” and 1573a “Tomato Leaves” from NIST, Gaithersburg, USA and n° 62 “Olive Leaves” from BCR, Bruxelles, Belgium) were used. Results of each batch of 24 samples were accepted only if data obtained from the certified reference materials were within the uncertainty range of the certified values (intervals with 95% confidence). Analytical precision from 6 replicate of certified reference materials, subjected to the same procedure as the wood samples, ranged from 2% for Cd to 15% for As. Concentrations of elements were expressed as mg/kg on a dry weight basis.

## 3 Results

Collected tree cores dated back to as early as the year 1942 for linden, 1960 for chestnut, and 1978 for pine and maple. In **Table 1** the overall concentrations of Hg and the other trace elements determined in tree ring core segments were reported. Being always below detection limits, Sb data is not included in the table. Among the analyzed elements, As, Cd and Co were very low in tree rings from Abbadia S.S., with median concentrations of 0.03 – 0.04 mg/kg or below the DL, irrespectively of the analyzed species. Also, Tl concentrations were generally negligible except for linden and maple cores, which showed concentrations up to 0.14-0.15 mg/kg. Overall Hg concentrations in core segments of tree species were considerable as they varied from the DL (0.05 mg/kg) up to the maximum level of 3.37 mg/kg found in the linden tree core.

**Table 1:** Mercury and PTEs concentrations (median, min-max; mg/kg) in tree rings from stands of four species (*A. pseudoplatanus*, n=11; *P. nigra*, n=9; *C. sativa*, n=13; *T. cordata*, n=27) collected from the urban area of Abbadia S.S.

	Maple (*A. pseudoplatanus*)		Pine (*P. nigra*)		Chestnut (*C. sativa*)			Linden (*T. cordata*)
	median	min-max	median	min-max	median	min-max	median	min-max
Hg	<DL		0.05	<DL-0.47	0.06	<DL-1.01	1.39	0.16-3.37
As	0.03	<DL-0.05	<DL		<DL		0.03	<DL-0.04
B	5.09	3.64-8.94	3.49	2.84-3.73	7.17	5.92-12.5	7.54	5.83-8.15
Cd	<DL		<DL		<DL		0.03	0.03-0.04
Co	<DL		0.03	<DL-0.03	0.04	0.03-0.07	0.03	<DL-0.20
Cr	0.08	0.01-3.26	0.68	0.20-2.53	2.27	1.10-5.39	1.57	0.44-18.4
Cu	0.71	0.40-1.99	0.66	0.56-1.12	1.14	0.90-7.08	1.22	1.09-2.75
Mn	0.39	0.22-1.18	2.23	1.85-3.98	2.84	1.56-6.04	2.08	1.21-4.41
Ni	0.06	0.02-0.63	0.25	0.10-0.92	0.54	0.34-1.12	0.52	0.20-5.73
Pb	0.03	<DL-0.07	0.03	<DL-0.04	0.09	0.05-0.18	0.17	0.08-0.25
Tl	0.05	<DL-0.15	<DL		<DL		0.06	0.03-0.14
V	0.03	<DL-0.10	0.03	<DL-0.05	0.05	<DL-0.23	0.03	<DL-0.43
Zn	1.78	1.12-14.5	5.90	4.79-8.95	23.2	11.6-39.3	4.95	2.57-25.7

DL, Detection limits.

**Figure 2:**
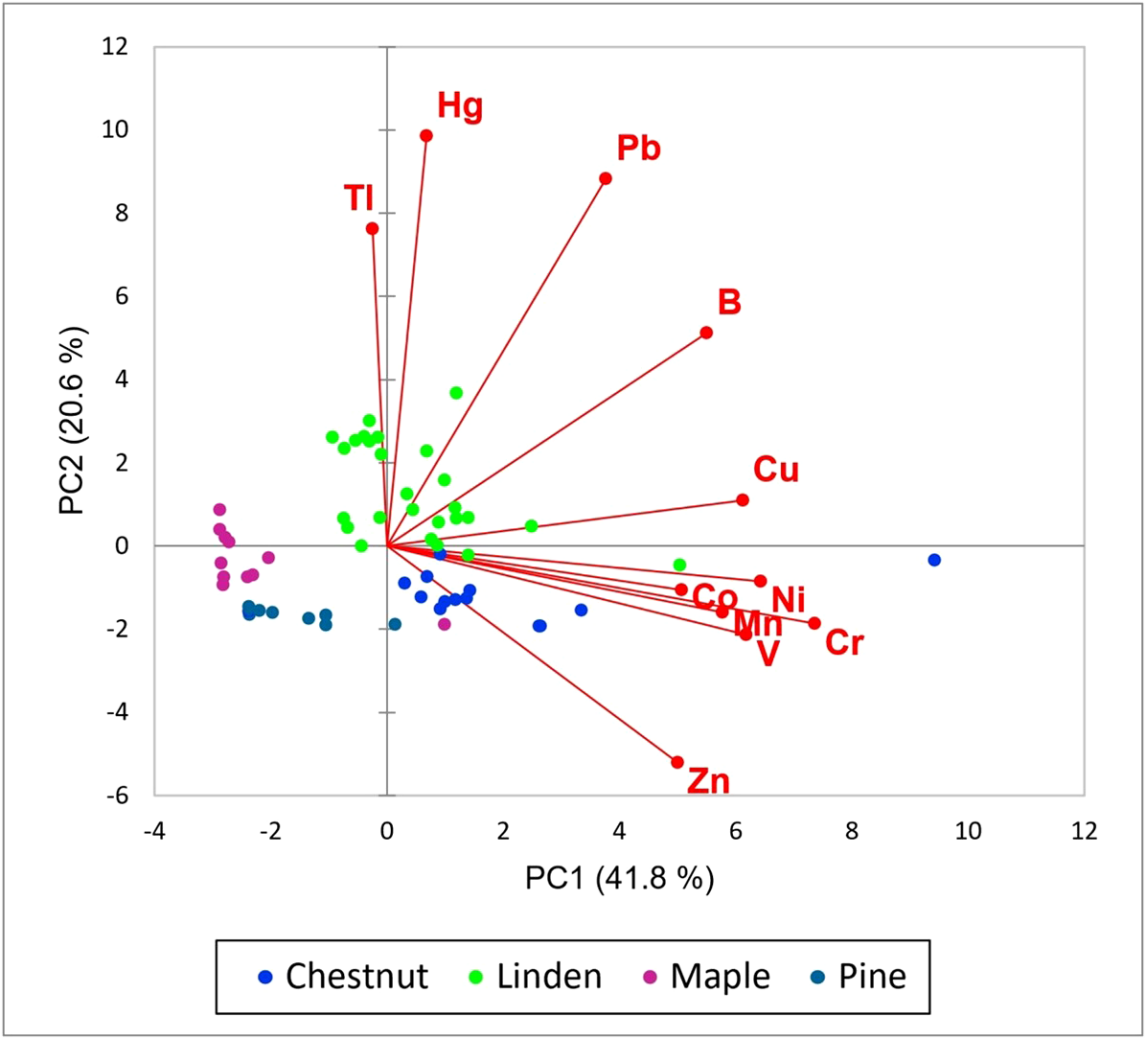
Projections of concentration data from tree ring segments in the vector space defined by PCA.

**Figure 3:**
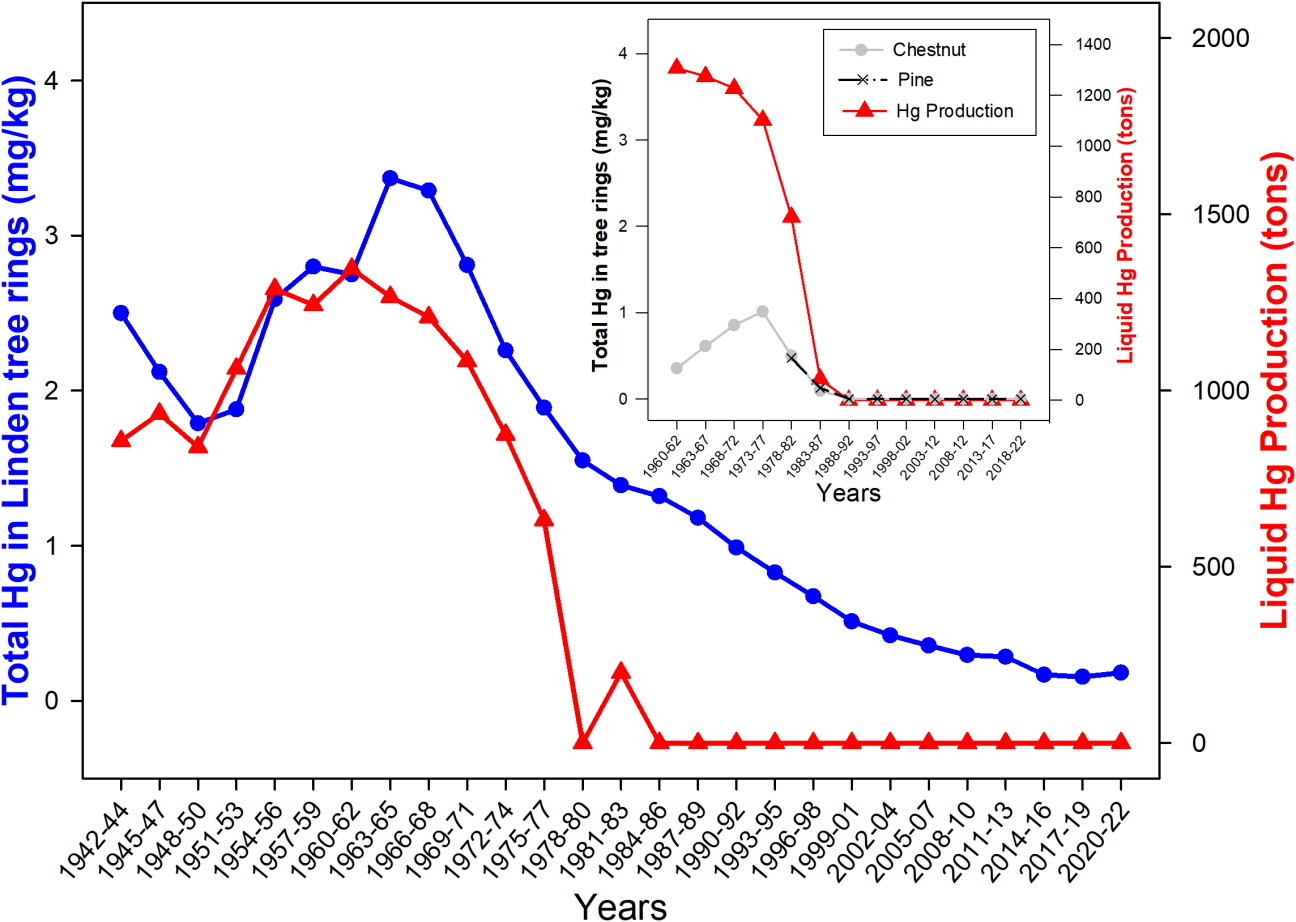
Total Hg concentrations in core segments collected from the linden tree (3-years segments) and from chestnut and pine trees (5-years segments) compared with the trend of liquid Hg tons produced in the mining district of Mt. Amiata (years 1942-2022); Hg produced quantities (Segreto, 1991; Forconi, 2011) were averaged for the corresponding time intervals of the segments.

The Principal Component Analysis (PCA) applied to the transformed and centered elements’ datasets singled out three principal components (PCs) with eigenvalues greater than 1 (Kaiser criterion). Sb, Cd, and As were excluded from the analysis, since data of these elements were mostly (> 60%) below the detection limits. The first two components (total variance 62.4%) of elements concentrations are shown as biplotted vectors together with individual data (**Figure 2**). Most variables (and especially Cr, Ni, V, and Cu) were correlated with the first PC, while PC2 had the highest loadings for Hg, Tl, and Pb. Despite contrasting Hg concentrations in different cores, Hg showed similar temporal trends. In **Figure 3** Hg concentrations in tree rings of linden, chestnut and pine trees are compared with the data of liquid Hg manufactured in the mining district of Mt. Amiata, including the largest contribution from Abbadia S.S. mining complex (Segreto, 1991; Forconi, 2011).

## 4 Discussion

Our findings showed a consistent relationship between the trend of Hg accumulation in wood and the metallurgical activity, taken as a reliable proxy of GEM emissions from the mining site (**Figure 3**). It has been estimated that approx. 8,000 tons of Hg have been released into the atmosphere by the metallurgical plants of Abbadia S.S. between 1943 and 1973 (Bombace et al., 1973). The highest Hg concentrations were found in tree rings from just after WWII and the 1960s. After this period of intense industrial activity, the mine experienced years of uninterrupted crisis which led to an unrelenting decrease in production, up to the complete closure in 1984. Hg contents in tree rings reflected well the reduction of Hg emissions linked to the crisis of the 1970s and indicated the actual levels of pollution, attributable to the abandoned mining-smelting plants (decade 2012-2022), currently under clean up for a complete remediation (Monaci et al., 2023). Linden tree data (**Figure 3**) show current levels of atmospheric Hg emission from the Abbadia S.S. mine site are at least twenty times lower than those of the years when the plant was fully operational. The temporal trends of chestnut and pine data generally agree with those observed for linden (albeit the comparison is only starting from the 1960s for chestnut and from the late 1970s for pine) and the progressive reduction of the activities at Abbadia S.S. mine of this period is reflected by decreasing of Hg contents in tree rings of all species. Concentrations of Hg in chestnut and pine for corresponding timespans (i.e. 1978-82 and 1983-87 segments; **Figure 3**) are almost identical, while they are remarkably lower (by a factor 3, at least) than those in corresponding segments of linden tree. Species-specific differences influencing stomatal uptake of Hg and its subsequent phloem translocation, including the expected phloem-to-xylem translocation, as well as the different duration of the vegetation period are thought the main factors that ultimately affect the levels of this metal in tree rings (Gačnik and Gustin, 2023). With regard to Hg assimilation in plant leaves, Pleijel et al. (2021) found large species-specific differences and a negative relationship between leaf-area-based Hg concentration and specific leaf area (SLA), while mass-based Hg concentration featured a somewhat weaker positive relationship with SLA. In this study, however, ecophysiological differences in Hg assimilation and retention among the different tree species (one conifer and three angiosperms) do not appear as crucial as the locations of the sampled stands. As it can be observed in **Figure 1** from the GEM dispersion around Abbadia S.S. mine, the linden tree is likely subjected to high atmospheric concentrations of Hg, being downwind of the metallurgical facilities. This is not the case for the chestnut and pine trees that, despite being close (~ 500 m) to the source of contamination, appear less affected by GEM contamination. In a comprehensive study conducted in the Mt. Amiata area, Fornasaro et al. (2023) collected stem discs from chestnuts located at ~2.5 km to the North-west of Abbadia S.S. mine, whose rings had much lower Hg concentrations (9.3-237 ng/g) than those of the chestnut tree of our study. In continuity with the present preliminary survey, further dendrochemical analyses of different species are in progress in the area to extend temporal trends to pre-industrial times and compare Hg contamination with the results of a long-term monitoring, currently under process since 2015, by using GEM passive samplers and *in situ* and transplanted lichens (Monaci et al., 2022).

PCA analysis allowed for distinguishing Hg, Tl, and Pb from the other PTEs, suggesting a common origin of these elements’ contents in tree rings. Several studies demonstrated the accumulation pathway via foliage of PTEs emitted by various industrial activities (Wright et al., 2014; Odabasi et al., 2016; Austruy et al., 2019; Arteau et al., 2020; Wang et al., 2022) and, in a first attempt to contextualize our data, Tl and Pb may have had environmental behaviors like those of Hg, at least during ore smelting activities. Polymetallic sulfide mineralizations are present in association with cinnabar deposits of Mt. Amiata and pyrite (FeS_2_) is ubiquitous, and locally abundant (Rimondi et al., 2015). A survey on mineral transformations and partitioning during pyrite ores roasting in the Chinese sulfuric acid plant of Yunfu pointed out that sulfide-bound Cd, Tl, and Pb, as semi-volatile elements in combustion processes, are significantly vaporized (completely in the case of Tl), before their gaseous species adsorb or condense on fine particles in the flue gas (Yang et al., 2009). The feedstock of this plant comes from the nearby Yunfu pyrite mine, the object of a more recent study on environmental pollution around it, where PTEs in needles, tree rings and soils of selected pine trees were measured; the data showed a strong positive correlation between Tl and Pb in soils and PCA analysis confirmed that Tl, Pb, Cd, and As in tree rings were mostly derived from pine needles (Wang et al., 2022). Similarly, the comparison of chemical and isotopic composition of tree rings and soils from a mining-smelting area of the Copperbelt province in Zambia has been found effective to identify the source of Pb in wood from the interception (foliar, bark) uptake of this metal by trees (Mihaljevič et al., 2011). On the other hand, depositions of atmospheric emissions from smelters can induce pedogeochemical modifications, such as acidification, that increase metal(loid)s bioavailability from soil and claim a more in-depth investigation of the complex processes driving PTEs from emissions to their corresponding recording by trees (Aznar et al., 2008; Arteau et al., 2020; Binda et al., 2021; Wang et al., 2022). In these circumstances, correlations between concentrations of gaseous atmopheric elements, such as Hg, with other PTEs in tree rings may also result from a combination of foliar and root uptake (Vaněk et al., 2011). Mercury concentrations in soils of Abbadia S.S. mine and surrounding areas have been characterized by Protano and Nannoni (2018), thus providing useful reference for assessing possible lithogenic contribution to contamination. To our knowledge, there are no available background values established for Pb and Tl in Abbadia S.S. or in the Mt. Amiata area that can be used for similar evaluations. This despite metall(oid)s pollution in the Mt. Amiata area (Hg and As, in particular) has recently become a matter of concern for the public health authorities, due to epidemiological anomalies in mortality and morbidity rates in residents (Minichilli et al., 2012; Bustaffa et al., 2017; Profili et al., 2018) exposed to the local contamination sources (former mining sites and operational geo-thermoelectric power plants). In a recent human biomonitoring survey, where a list of several selected PTEs was considered, exposure to Tl proved to be the novel critical feature of the area, potentially associated with geothermal exploitation but with still uncertain exposure pathways (Nuvolone et al., 2023). Keeping in mind the limitations of our pilot study, we can speculate about the possibility to include Pb in the parameter list of the next human biomonitoring campaigns.

Finally, despite significant gaps in the knowledge of the ecophysiological mechanisms driving the PTEs cycling in trees and their transport and accumulation in woody tissues, tree ring analysis is a promising tool to infer past and present impacts on environmental quality and to evaluate the resulting human exposure.

## Data availability statement

The raw data supporting the conclusions of this article will be made available by the authors, without undue reservation.

## Author contributions

DB: Conceptualization, Writing – original draft, Writing – review & editing, Investigation. SA: Investigation, Writing – review & editing. JF: Writing – review & editing, Investigation. SL: Writing – review & editing, Investigation. FM: Writing – review & editing, Conceptualization, Funding acquisition, Visualization, Writing – original draft, Investigation.
